# Youth perspectives on sexually transmitted infections and sexual health in Northern Canada and implications for public health practice

**DOI:** 10.3402/ijch.v75.30706

**Published:** 2016-12-09

**Authors:** Gwen Healey

**Affiliations:** Qaujigiartiit Health Research Centre, Iqaluit, NU, Canada and Northern Ontario School of Medicine

**Keywords:** Inuit, adolescent, sexual health, sexual health education, parent–adolescent relationships, determinants of health

## Abstract

**Objective:**

High rates of sexually transmitted infections in the Arctic have been a focus of recent research, and youth are believed to be at greatest risk of infection. Little research has focused on understanding youth perspectives on sexual health. The goal of this study was to collect the perspectives of youth in Nunavut on sexual health and relationships with the intent of informing public health practice.

**Method:**

This qualitative research study was conducted within an Indigenous knowledge framework with a focus on Inuit ways of knowing. Data were collected through face-to-face interviews in three Nunavut communities with 17 youth between the ages of 14 and 19 years. Participants were asked open-ended questions about their experiences talking about sexual health and relationships with their family, peers, teachers or others in the community.

**Results:**

There are four key findings, which are important for public health: (a) Parents/caregivers are the preferred source of knowledge about sexual health and relationships among youth respondents; (b) youth did not report using the Internet for sexual health information; (c) youth related sexual decision-making to the broader community context and determinants of health, such as poverty; and (d) youth discussed sexual health in terms of desire and love, which is an aspect of sexual health often omitted from the discourse.

**Implications and contribution:**

The youth in this study articulated perspectives on sexual health, which are largely neglected in current public health practice in the North. The findings from this study underscore the important role of community-led participatory research in contributing to our understanding of the public health challenges in our communities today, and provide direction for future interventions and research.

High rates of sexually transmitted infections (STIs) in the Arctic have been a focus of recent research, and young people are believed to be at greatest risk of infection. In a study of STIs in the North American Arctic and Greenland, Gesink Law, Rink, Mulvad and Koch (1) found that Arctic regions consistently reported higher rates of infection than their southern counterparts. In 2006, Alaska reported high rates of chlamydial infection (715 cases/100,000 population) compared with the United States as a whole; northern Canada reported high rates of chlamydial infection (1,693 cases/100,000) and gonorrhea (247 cases/100,000) compared with southern Canada; and Greenland consistently reported the highest rates of chlamydial infection (5,543 cases/100,000) and gonorrhea (1,738 cases/100,000) in the Arctic (1). Rates were high for both men and women, although the highest incidence of infection was predominantly reported for young women in their early 20s (1). The authors noted that community-based participatory research was needed to improve sexual health in Arctic communities and engage community members in the collection of data and the development of appropriate interventions. A recent study in Greenland found the presence of an emerging STI, *Mycoplasma genitalium* with symptoms similar to those for *Chlamydia trachomatis* and *Neisseria gonorrhoeae* (2).

Nunavut is Canada's third northern territory, which was created in 1999 (3). There are 25 communities in Nunavut, ranging in size from a population of 110 to a population of 7,000. The entire population of Nunavut in 2014 was 36,100, of whom approximately 85% were Inuit (4). Nunavut has a very young population; in 2006, 53% of the Nunavut population was consisted of those 24 years of age and younger (4). In 2013, Nunavut's rates of chlamydia, gonorrhea and syphilis were more than 10 times higher than that of Canada, and still remained high in 2014/15. In a report of STIs in Nunavut from 2007 to 2014, Nunavut reported consistently high incidence of chlamydia (between 3,400 and 3,772 per 100,000 people) and gonorrhea (between 900 and 1,588 per 100,000 people) compared to Canadians (259/100,000 and 33/100,000, respectively) (5,6). Nunavut saw an increase in syphilis beginning in 2012, with 94 reported cases in 2014 (7). The average age of cases in 2014 was 31 years old, with more female cases (56%). No new HIV cases were reported in Nunavut from 2007 to 2014 (7).

In a review of the literature, a number of issues related to sexual health in northern communities were highlighted, including the role of family in sexual health education (8–17); the influence of the Internet and other media (18,19); teen pregnancy and custom adoption (11,20–22); history of sexual abuse (23–30); alcohol and substance use and its role in emotional and behavioural regulation as well as increased risk for unprotected sex and sexual violence (31–39); mental well-being (40,41); and knowledge about physiology, reproduction, sexual health and well-being (18,42,43). Westernization and colonization have been identified in the literature as negative influences on sexual health because of the loss of the accumulated wisdom and knowledge of Inuit regarding the life cycle, reproductive health and family planning which are no longer shared by Inuit families, and contributed to many Inuit parents and grandparents no longer feeling competent to instruct their children (1,18,26,27).

High rates of STIs in the Arctic have been a focus of recent research, and youth are believed to be at greatest risk of infection (26,27,44,45). There is a paucity of research that has focused on understanding youth perspectives on sexual health. The purpose of this study was to investigate the perspectives of young Inuit in Nunavut, Canada, on the topic of STIs, and sexual health in general, to better understand the determinants of the high rates of infection among this age group and contribute to evidence-based public health practice.

## Method

This qualitative research study was conducted within an Indigenous knowledge framework with a focus on Inuit ways of knowing, specifically the *Piliriqatigiinniq* Partnership Community Health Research Model (46). The model highlights five Inuit concepts, which informed the research approach: *Piliriqatigiinniq* (the concept of working together for the common good), *Pittiarniq* (the concept of being good or kind), *Inuuqatigiittiarniq* (the concept of being respectful of others), *Unikkaaqatigiinniq* (the philosophy of storytelling and/or the power and meaning of story) and *Iqqaumaqatigiinniq* (the concept that ideas or thoughts may come into “one”). The study was designed to respond to a community request, and it was implemented collaboratively with community wellness centres, emulating the concepts of *Piliriqatigiinniq* and *Inuuqatigiittiarniq*. Ethical approval was obtained from the University of Toronto and a research license was granted by the Nunavut Research Institute. A narrative was provided addressing ethical concerns commonly raised by Nunavut communities, which was discussed with participants before engaging in the study. This open and reciprocal approach was part of demonstrating goodness or kindness, *Pittiarniq*. Participants were invited to participate through the community wellness centres. Data were collected through face-to-face interviews in three Nunavut communities with 17 youth between the ages of 16 and 19 years, including one 14-year-old girl who accompanied her parent to the interview. Interviews were conducted in a comfortable setting chosen by the participant, recorded with permission and transcribed verbatim. With respect to the concept of *Unikkaqqatigiiniq*, participants were asked open-ended questions about their stories and experiences talking about sexual health and relationships with their family, peers, teachers or others in the community. Data were analysed by the researcher through a process of immersion and crystallization (47), which is a process analogous to the concept of *Iqqaumaqatigiinniq*, “all knowing coming into one.” Through a process of listening to interviews, reading and re-reading transcripts and stories, themes crystallized in the data. The researcher presented the themes to community advisors for the project. A rigorous, respectful and mindful process was followed for the data analysis, which included the comparison of findings to the known literature on the topic (48), reflexivity and bracketing of researcher perspectives before and during the study (49,50), an iterative data collection and analysis process (51) and discussion of findings with community advisors (51).

## Results

Four key findings were identified in the analysis: (a) Parents/caregivers are the preferred source of knowledge about sexual health and relationships among youth respondents; (b) youth did not report using the Internet for sexual health information; (c) youth related sexual decision-making to the broader community context and determinants of health, such as poverty; and (d) youth discussed sexual health in terms of desire and love, which is an aspect of sexual health often omitted from the discourse.

First, the participants’ definition of “sexual health” is discussed. Then results are presented under the following headings: (a) Preferred sources of information about sexual health, (b) Perspectives on sex and relationships and (c) Youth sexual health and relationships in the community context.

### Participants’ definition of “sexual health”

The majority of the youth (16 of 17) in this study defined sexual health using terms they had learnt in school. For example, they spoke primarily of sexual health in terms of condom use and STIs. Most of the youth (15 of 17) reported knowing where to get condoms, how to use them and indicated that they had learnt about sexual health in “family life” or “health” class in school. Respondents who did not know how to use condoms or where to get them were also not regular schools attenders.

### Preferred sources of information about sexual health

When asked whether they would like to know more about sexual health, 14 of 17 youth indicated that they knew enough and did not need more information, even though few of the respondents were able to articulate an understanding of STIs beyond the basic understanding that there were illnesses one could contract through sex. Some youth knew about HIV/AIDS, and only one youth knew about chlamydia or gonorrhea, which are the two most prevalent STIs in Nunavut.

Only one out of the 17 youth interviewed indicated that they used the Internet to learn more about sexual health. One youth participant stated,

We only use the Internet for Facebook. – Young woman – community 1

Youth were asked from whom or how they would prefer to learn about sexual health and romantic relationships. Examples included a nurse, a teacher, the Internet, caregivers/parents or some other means. Of the 17 youth, 16 indicated caregivers/parents. Youth shared stories about parents who struggled with alcohol use; parents who were separated or separating; but highlighted that even if they did not have a positive relationship with their parent at that time, they would still prefer to learn about sexual health and relationships from them than from any other source.

### Perspectives on sex and relationships

When asked about why they themselves or other young people in their community were engaging in sex, the reasons included boredom, peer pressure, too much free time, hopelessness stemming from unemployment and poverty in the community, desire to start a family and desire and love for their partner.

… [They are having sex]. because it's fun … uh-hm, they want to have kids, too …. – Young woman – community 1.

Um, I found that a lot of the guys wanted to know more about sex than the health aspect. They wanted to know like, what's the different experiences for boys and girls – like, do boys find it more important to have sex or do girls? … it has different meanings for different people. Like some people see it as a strengthener for relationships; some people see it as a form of pain relief and some people see it as just the act to have to conceive children. To a lot of youth, sex … is just an activity, like it has no meaning. And there's no long term commitment unless something happens like … pregnancy. – Young man – community 2

One participant underscored the importance of ensuring youth understand that they have a choice.

You know sexual health is a choice. You can't make someone use a condom, you can't make them use birth control. It has to be their own decision. And I find that when [I'm] talking to [other] youth, that's the most important part. You don't say “use condoms.” You say “if you want to have sex, it's a good idea to use condoms”… Emphasize the choice. – Young man – community 2

### Youth sexual health and relationships in the community context

In one community, youth participants talked about the role of poverty in their community in response to a question about how youth want to be supported to make safe sexual decisions.

Yeah. Um, they need help sometimes … No food [at home]. All that. Yeah. Some – are poor. Like … Some people … go on the radio or CB [radio] to borrow milk or food. For their kids. I heard it a lot. And they have lots of kids who are hungry. – Young woman – community 1

In this community, youth shared a sense of hopelessness for their own future because they did not believe that they would be able to get paid employment. The high rate of unemployment was largely because there were more people in the community than there were jobs.

## Discussion

There are four findings from the analysis of the data that are important for public health: (a) Youth identified that parents/caregivers were the preferred source of knowledge about sexual health and relationships; (b) youth did not report using the Internet for sexual health information; (c) youth related sexual decision-making to the broader community context and determinants of health, such as poverty; and (d) youth discussed sexual health in terms of fun, desire and love, which are aspects of sexual health and behaviour often omitted from the discourse. Each of these points is discussed further below.

First, youth participants indicated their preferred sources of knowledge about sexual health and relationships to be parents/caregivers, even if they had not discussed sexual health with their parents/caregivers previously. They almost universally rejected the school system, the nurse/community health representative and the Internet as preferred sources of knowledge about sexual health and relationships. The protective benefits of parent–adolescent communication about sexual health are well known (8,10,27,52). In a youth sexual health study in Greenland, Rink, Montgomery-Anderson and Anastario found that the influence of having a parent/guardian to speak with about topics related to sex, including the consequences of pregnancy, was a key protective factor in reducing STIs among Greenlandic youth (44).

Second, youth participants indicated that they do not use the Internet to find information about sexual health. This is an important finding because significant financial resources have been put into the development and launch of online sexual health promotion resources for youth in the past several years. Youth participants noted that the primary reason for using the Internet was to use Facebook for entertainment and communication. Similar findings were reported by Jones and Biddlecom (53) in a study of adolescents’ use of the Internet for sexual health information. The authors found that students were more likely to rely on and had greater trust in what the researchers described as “traditional sexuality education sources”, such as school, family members and friends, and were wary of sexual health information on the Internet (53). However, this finding counters a result from a study with Alaska Native and non-Alaska Native youth in which they preferred to receive messages via the Internet and schools (54). Many respondents indicated that they knew enough about sexual health already, which may be why they were not pursuing additional information on the Internet. Most households in Nunavut do not have private Internet access in their home; therefore, youth usually access the Internet through the school or community access programme sites, which are public spaces with controls in place on the web browsers. The intention of these controls is to protect users from sexually exploitative content, such as pornography, and they may also block content that is meant to be educational, such as sexual health education websites.

Third, youth related their understanding of sexual health and relationships to the community context, specifically the issue of poverty and hardship. In a discussion about supports needed to make safe sexual decisions, youth talked about hunger and the number of people in the community who require assistance to feed their families and survive day-to-day. Their responses indicated that the basic needs of families in the community must be met before youth can participate in safe sexual decision-making. This is similar to some of the ideas conveyed in the Inuit hierarchy of human needs (55–57), adapted by Inuit elders from Maslow's hierarchy of needs, which described a theory of human behaviour based on a series of linear steps, the first being the meeting of physiological needs and ensuring safety of body, health and family. Youth participants described the need for physiological requirements to be met in order to make safe sexual decisions. Youth participants also described a sense of hopelessness for their future and what they perceived to be a lack of economic or scholarly opportunities in their community in the context of sexual decision-making among their peers. In a review of the effects of social determinants of health on adolescents, Viner et al. (58) found the strongest determinants of adolescent health worldwide were structural factors such as national wealth, income inequality and access to education. In addition, safe and supportive families and safe and supportive schools, together with positive and supportive peers, were identified as crucial factors for helping young people develop to their full potential and attain the best health in the transition to adulthood (58). The perspectives shared by the youth participants in the present study add to this literature on the impact of social determinants on adolescents.

Lastly, youth discussed sexual health and relationships in terms of feelings of desire and love for their partners, which are important components to the dialogue about sexual health relationships that are often neglected in the literature. Fine (59) argued that the rhetoric surrounding sex education and school-based health clinics has done little to enhance the development of healthy sexual attitudes and responsible sexual behaviour in adolescents. The author advocated for a “discourse of desire,” which could contribute to the intellectual, social and sexual empowerment of young women (59). Fine (59) felt the consequences of failing to develop such a discourse included teenage pregnancy, increased dropout rates and sexual victimization. Fine's research focussed on female empowerment, and more research on sexual empowerment among young men, women and transgender youth in the North is needed. In a more recent study, Shoveller and Johnson (60) argued that significant public health attention has focused on the “problems” of youth sexual behaviour, and empirical public health research in this area has attempted to account for mostly negative sexual health outcomes (e.g. STIs and teenage pregnancies) by examining individual characteristics and risk-taking behaviour. Public health practice has followed suit, focusing primarily on modifying sexual risk behaviour and lifestyle “choices” (60). In the present study, the youth perspectives on love and desire as part of the definition of sexual health send a clear message to public health practitioners and researchers - that there are other lenses through which the topic of sexual health and relationships, and positive youth sexual behaviour, can be viewed.

## Implications and contribution

Public health practice has much to learn from the voices of young people. The youth in this study articulated perspectives on sexual health, which are largely absent from the literature and neglected in current public health practice in the North. Public health practice can build on these findings by implementing interventions to address the concerns of the youth population. Such interventions can include parent–adolescent education initiatives, and should be embedded in a holistic approach, which includes a social determinant of health lens and consideration of physical, emotional, mental and spiritual health. The use of *Piliriqatigiinniq* model allowed for the fostering of relationships within the communities that participated in the study (*Inuuqatigiitsiarniq*), and privileged the stories and voices of the youth in the method (*Unikkaaqatigiinniq*). As a result, the findings from the study add completely new knowledge to this area of study. The findings also underscore the important role of community-led participatory research in contributing to our understanding of the public health challenges in our Arctic communities today and provide direction for future interventions and research.
